# A Sustainable Multi-Objective Model for Capacitated-Electric-Vehicle-Routing-Problem Considering Hard and Soft Time Windows as Well as Partial Recharging

**DOI:** 10.3390/biomimetics9040242

**Published:** 2024-04-18

**Authors:** Amir Hossein Sheikh Azadi, Mohammad Khalilzadeh, Jurgita Antucheviciene, Ali Heidari, Amirhossein Soon

**Affiliations:** 1 School of Industrial Engineering, College of Engineering, University of Tehran, Tehran 1417614411, Iran; amir1377623@gmail.com; 2 Industrial Engineering Department, Faculty of Engineering and Natural Sciences, Istinye University, Sarıyer, Istanbul 34396, Turkey; mohammed.khalilzadeh@istinye.edu.tr; 3 Department of Construction Management and Real Estate, Vilnius Gediminas Technical University, 10223 Vilnius, Lithuania; 4 Department of Industrial Engineering, Iran University of Science and Technology, Tehran 1684613114, Iran; 5 Faculty of Engineering, University of Hormozgan, Bandar Abbas 7916193145, Iran; amir.soon1376@gmail.com

**Keywords:** capacitated EVRP, sustainability, partial recharging, hard and soft time windows, epsilon-constraint, nature-inspired algorithms

## Abstract

Due to the high pollution of the transportation sector, nowadays the role of electric vehicles has been noticed more and more by governments, organizations, and environmentally friendly people. On the other hand, the problem of electric vehicle routing (EVRP) has been widely studied in recent years. This paper deals with an extended version of EVRP, in which electric vehicles (EVs) deliver goods to customers. The limited battery capacity of EVs causes their operational domains to be less than those of gasoline vehicles. For this purpose, several charging stations are considered in this study for EVs. In addition, depending on the operational domain, a full charge may not be needed, which reduces the operation time. Therefore, partial recharging is also taken into account in the present research. This problem is formulated as a multi-objective integer linear programming model, whose objective functions include economic, environmental, and social aspects. Then, the preemptive fuzzy goal programming method (PFGP) is exploited as an exact method to solve small-sized problems. Also, two hybrid meta-heuristic algorithms inspired by nature, including MOSA, MOGWO, MOPSO, and NSGAII_TLBO, are utilized to solve large-sized problems. The results obtained from solving the numerous test problems demonstrate that the hybrid meta-heuristic algorithm can provide efficient solutions in terms of quality and non-dominated solutions in all test problems. In addition, the performance of the algorithms was compared in terms of four indexes: time, MID, MOCV, and HV. Moreover, statistical analysis is performed to investigate whether there is a significant difference between the performance of the algorithms. The results indicate that the MOSA algorithm performs better in terms of the time index. On the other hand, the NSGA-II-TLBO algorithm outperforms in terms of the MID, MOCV, and HV indexes.

## 1. Introduction

In recent decades, the topic of logistics has received a lot of attention from service and production companies. Logistics management means planning, organizing, leading, coordinating, and controlling logistics activities and implementing them in the best way in order to improve the efficiency of the logistics system and increase profits. The logistics distribution system serves as a bridge between the supply and demand points in the logistics network, and its purpose is to deliver materials, goods, or products from the supply points to the demand points at the right time and in the right size, keeping in mind the economic conditions. The transportation sector in the distribution of logistics accounts for the highest cost, and cost management in this sector is highly sensitive because the cost of product transportation significantly affects the final product price. As a result, minimizing the costs of the entire distribution system is among the primary objectives and goals of the logistics companies. Nowadays, various logistics firms seek to effectively plan for decreasing their operational costs and rendering appropriate services to their customers in order to increase customer satisfaction and survive in this competitive global market. Several documents and evidence demonstrate that optimizing the distribution system has led to significantly decreasing the costs of transportation [1]. Moreover, it should be noted that losing only 5% of customers will result in reducing the income of the logistics system between 25% and 85%, which is pertinent to the nature of the industry. Customer satisfaction as an essential requirement for retaining customers may enhance the life cycle of the customer [2]. These issues have resulted in the significant development of the vehicle routing problem (VRP). VRP directly affects the level of customer service and distribution costs. Distribution centers and customers are known as the main components of logistics distribution, and routing is between distribution centers and customers.

Moreover, the Sustainable Development Goals (SDG) of the United Nations (UN) require world-wide countries to pay more attention to the environment. To this end, with the increase in urbanization, tremendous efforts have been made in the transportation industry to address confined fossil fuels and natural resources, climate change, and environmental issues. Decreasing air pollution compels the logistics industry, which is accused of 20% of the entire air pollution. [3]. As a result, developing environmentally friendly transportation and logistics systems has been addressed in the last decade because of transportation operations that affect environmental pollution as well as global warming. There are also growing social and governmental concerns about environmental issues in the form of new rules, regulations, and laws, as well as social incentives leading to the proposal of novel distribution and transportation models [4]. Various studies and research works have been conducted in the field of green transportation and logistics, aiming to enhance transportation sustainability while considering social, environmental, and social problems. The use of EVs instead of traditional vehicles and their impacts on the environment is a major topic for these studies on green transportation and distribution [5,6,7]. Several developed countries have been cooperating to produce environmentally friendly vehicles ranging from small cars to large trucks in order to decrease greenhouse gas emissions and optimize fuel usage [8].

The above-mentioned problems explicitly show the importance and motivation of studying environmentally friendly logistics and transportation systems. As a result, the aim of this paper is to address the use of EVs instead of vehicles that consume fossil fuels. The present study aims to develop a multi-objective mathematical programming model for the Capacitated Electric Vehicle Routing Problem (CEVRP), considering all sustainability aspects. In the proposed model, the capacity of EVs, the possibility of recharging, and time windows for availability are taken into account. In addition, the hard time window for customer service is assumed to increase customer satisfaction. It is presumed that there is a maximum time or deadline for customer service in order to return the EVs to the final (destination) point in such a way that EVs are available for re-serving in the upcoming time periods. Moreover, the early return of the EVs to the terminus point ultimately leads to enhanced customer service and customer satisfaction. The first objective function of the proposed model is to minimize the total cost, which is one of the most crucial factors for any logistics company, by choosing the optimal routes. On the other hand, the second objective function attempts to minimize the negative environmental effects by optimizing the amount of charging and loading EVs. On the contrary, the third objective function tries to maximize customer satisfaction by fulfilling customer demand within an appropriate time window. In order to solve this model and find the optimal solution, the EC exact method is employed for small-sized problems. Also, three MOSA, MOGWO, and NSGA-II-TLBO meta-heuristic algorithms are exploited to deal with large-sized problems, as the problem is HP-Hard. In addition, the impact of changing the main model parameters is analyzed on the results.

The research questions can be mentioned as follows:

1. What are the indicators of pollution reduction in the studied CVRP problem in this research?
2. What is the effect of the time window on reducing the emission of pollution by the green vehicle?
3. How is it possible to recharge green vehicles along the way?
4. What is the effect of using green vehicles with different capacities on reducing pollution?

It should be noted that the MOSA algorithm is an extension of SA that is inspired by the annealing process in metallurgy and is used to change the chemical and physical properties of a material. The MOGWO algorithm is an extended gray wolf optimization algorithm that is inspired by the behavior of a type of wolf called the gray wolf. It was simply created by studying and examining the characteristics and behaviors of gray wolves. The NSGA-II algorithm is one of the leading and most widely used algorithms for multi-objective optimization problems. This algorithm, which was inspired by the principles of natural selection and genetics, mimics the process of natural evolution to find optimal solutions to complex problems. The TLBO optimization algorithm, such as other meta-heuristic algorithms, is a population-based algorithm derived from nature and works based on the influence of a teacher on learning in the classroom. In the present research, the NSGA-II algorithm is combined with the TLBO algorithm.

The remainder of this paper is organized as follows: The literature is thoroughly reviewed in Section 2. Section 3 proposes the sustainable multi-objective optimization model. Subsequently, Section 4 elaborates on the solution approaches. Then, the proposed optimization model is validated in Section 5. The efficiency of NSGA-II-TLBO is compared with two recent meta-heuristic algorithms, MOSA and MOGWO, and the findings are supplied in Section 6. At the end, a conclusion and suggestions for further research are presented in Section 7.

## 2. Literature Review

The Vehicle Routing Problem (VRP) as a substantial problem in goods distribution management has attracted the attention of numerous practitioners and researchers. The VRP problem is categorized into several classifications according to the objective functions and constraints of the distribution systems. This diversification leads to a broad range of problems with various structural and operational features. Since the VRP problem has been extensively investigated by several scholars, it is intricate to examine the improvement of all its classifications during the last decade. In this section, a review of the relevant studies on Capacitated VRP (CVRP) and Electric VRP (EVRP) is presented.

VRP was introduced by Golden et al. in 1977 for the first time. Since then, this problem has been widely investigated [9]. Solomon [10] proposed the concept of a time window. Then, Erdoğan, and Miller-Hooks [11] presented the Green VRP (GVRP), assuming that the vehicles are fully refueled with a constant refueling duration in the defined stations on the predetermined routes. They developed a single-objective mixed integer linear programming (MILP) mathematical model for minimizing the route traversed by the non-capacitated vehicles, considering the confined tour distance and distinctive duration. Subsequently, Felipe et al. [12] studied GVRP considering partial recharging and diverse technologies, in which the technologies of the charging stations are different and green vehicles can be partially recharged. They designed a MILP optimization model aiming to minimize the total costs of refueling and the total distance traversed, and they solved the model using the Simulated Annealing (SA) meta-heuristic algorithm with a local search heuristic algorithm.

Schneider et al. [13] investigated EVRP, considering recharging stations and time windows, and solved numerous problem instances. Then, Koç and Karaoglan [14] introduced a VRP model and employed the branch-and-cut exact method and SA to find better upper bounds and initial solutions. Subsequently, Xiao and Konak [15] developed a MIP model for the scheduling of CVRP, assuming the time window for customer service and routes’ traffic. Followingly, Leggieri and Haouari [16] formulated both capacitated and non-capacitated VRPs.

Sedighizadeh and Mazaheripour [17] introduced an optimization model with multiple objectives for CVRP, including the objective functions of minimizing the number of vehicles, travel duration, and cost, and maximizing the discrepancy between the earliest and latest times for all customers. They assumed hard time windows for serving and visiting customers and exploited a hybrid of the Particle Swarm Optimization (PSO) and Artificial Bee Colony (ABC) meta-heuristic algorithms as a solution approach. Then, Montoya et al. [18] presented an optimization model for EVRP considering the recharging durations of EVs. Later on, Keskin and Catay [19] applied an Adaptive Large Neighborhood Search (ALNS) meta-heuristic algorithm for a type of GVRP, considering various types of vehicle recharging. Zhang et al. [20] computed the EVs’ energy consumption and exploited a meta-heuristic approach to deal with it. Then, Jie et al. [5] proposed a kind of two-echelon CEVRP, considering several assumptions such as diverse battery capacity, load capacity, and battery recharge cost. Finally, a hybrid meta-heuristic algorithm was exploited to tackle this problem. Subsequently, Mackrina et al. [21] investigated GVRP, considering two kinds of fossil fuel and EVs, as well as time windows and partial recharging. Basso et al. [22] designed a mathematical model for EVRP for calculating energy consumption, taking several assumptions such as topology, traffic, speed, and number of intersections into account. Xu et al. [23] addressed the capacitated green VRP considering soft time windows and time-varying vehicle speed and solved this problem by using the NSGA-II meta-heuristic algorithm.

Zhang et al. [24] proposed a fuzzy mathematical model for EVRP considering recharging stations and time windows and exploited the Adaptive Large Neighborhood Search (ALNS) meta-heuristic algorithm to solve the model. Lu et al. [25] investigated a time-dependent EVRP with the objective of cost minimization to specify the departure time and speed of the vehicle and dealt with this problem by employing a meta-heuristic algorithm named Iterated Variable Neighborhood Search (IVNS). Zulvia et al. [26] investigated the green VRP for perishable products to minimize costs and carbon emissions while maximizing customer satisfaction and solved it by implementing the many-objective gradient evolution (MOGE) meta-heuristic algorithm. Zhou et al. [27] proposed a novel collaborative multi-heterogeneous-depot electric vehicle routing problem with mixed time windows and battery swapping. Bahrami et al. [28] proposed a type of VRP in which the vehicles consume gasoline and electricity and solve the problem using the branch-and-price algorithm. Zhu et al. [29] developed an optimization model to decrease air pollution in the multi-depot EVRP and applied meta-heuristic algorithms as the solution approached. Mavrovouniotis et al. [30] proposed a mathematical programming model for EVRP and employed a meta-heuristic algorithm to solve it. Kozák et al. [31] presented a model for EVs.

Moreover, Froger et al. [32] designed a mathematical programming model for EVRP considering recharging stations. Furthermore, Futalef et al. [33] modeled the single-objective CEVRP and solved the model using the GA meta-heuristic algorithm. On the other hand, Gupta et al. [34] developed another optimization model with multiple objectives, considering multiple depots for minimizing fuel emissions in VRP, and solved it using a hybrid GA. Karakatič [35] introduced a multi-objective mathematical programming model for recharging EVs in CVRP in order to minimize stops as well as travel and recharging times and employed a Two-Layer GA for solving the problem. Arias-Londoño et al. [36] proposed a MILP model for CVRP, taking the last-mile delivery, battery capacity, and recharging station into account, and solved a test problem with the GAMS optimization software. Furthermore, Pan et al. [37] studied an urban CVRP considering time windows, travel and loading time, maximum trip duration, and vehicle multiple-trip aiming to reduce the total traveling distance. They also used a hybrid of ALNS and VND meta-heuristic algorithms to tackle the problem. Fan et al. [38] developed an integer model for multi-depot VRP considering different assumptions such as vehicle load, speed, and time-varying road gradient to minimize the total costs comprising fixed costs, penalty costs, and fuel costs. This problem was solved by using the combination of GA and VNS. The numerical problems demonstrated the effectiveness of their model and solution approach. Olgun et al. [39] investigated GVRP, considering customer pickup and delivery demand at the same time to minimize cost, and exploited a hyper-heuristic algorithm based on iterative local search (HH-ILS) to solve the problem. The numerical problems indicate the efficiency of this algorithm. Moreover, Moghdani et al. [40] conducted a systematic review of the literature on GVRP, considering different objective functions, uncertainty, and methodologies published from 2006 to 2019. They concluded that studies on GVRP are comparatively fresh and that significant improvements can be made in various areas. Hesam Sadati et al. [41] applied the Variable Tabu Neighborhood Search (VTNS) meta-heuristic algorithm to solve VRP with multiple depots and time windows. The findings display the effectiveness of the solution method. Wang et al. [42] studied VRP with multiple depots and multiple time periods for sharing logistics resources to minimize transportation cost, number of vehicles, and service waiting time and employed a hybrid NSGA-III meta-heuristic algorithm to solve the problem. They also implemented their model in a case study in China to validate the model and algorithm.

Xue et al. [43] studied the two-echelon dynamic VRP, which transforms customers with useless storage into satellite stations, aiming to minimize make-to-stock and operational costs. They exploited a hybrid of GA and TS to solve this problem. Finally, a case study in China was considered to demonstrate the model’s efficiency. Xiang et al. [44] implemented an Ant Colony Optimization meta-heuristic algorithm based on coverage diversity on a type of VRP to retain the variety of customers covered within routes in order to respond to new customer demands. Several test problems were also used to show the effectiveness of the model and algorithm. Li et al. [45] developed the Markov decision model to identify the optimal time allocation policy for the fleet of EVs and proposed the dynamic programming algorithm for solving this problem. They implemented the model using numerical examples for validation. Bruglieri et al. [46] suggested the Greedy Randomized Adaptive Search Procedure (GRASP) for solving a classification of the GVRP to minimize the total distance traveled. Erdem [47] proposed a sustainable MIP model with the goals of optimizing transportation operations and waste collection for an extension of EVRP in which EVs visit the waste bins and employed the Adaptive Variable Neighborhood Search (AVNS) algorithm for solving the problem. Wen et al. [48] exploited an improved ALNS meta-heuristic algorithm to solve GVRP with time windows and multiple depots. The results indicate the high accuracy and speed of the ALNS meta-heuristic algorithm.

Kuo et al. [49] modeled a VRP considering time windows and the objectives of minimizing costs and carbon emissions. They also applied the improved MOPSO meta-heuristic algorithm to solve this problem. The findings demonstrate the efficiency of MOPSO. Zhang et al. [50] introduced a type of CVRP considering stochastic demands. They used Monte Carlo simulation and scenario analysis to minimize the expected total logistics costs and employed a hybrid meta-heuristic algorithm based on Adaptive Tabu Search (ATS) to solve this problem. Wang and Zhao [51] investigated EVRP, considering the strategy for partial linear recharging, time window, and heterogeneous vehicles, and developed an efficient heuristic algorithm based on LNS to solve the problem. Moreover, Wang et al. [52] proposed a bi-objective non-linear model for EVRP considering multiple depots, shared charging stations, and time windows in order to minimize the number of EVs and total operational costs. They also suggested a hybrid of Multi-Objective GA and TS algorithms for solving this problem. The results reveal that the algorithms can considerably reduce operational costs. Furthermore, Xiao et al. [53] studied EVRP considering mixed backhauls and time windows and proposed the Diversity-Enhanced Memetic meta-heuristic algorithm (DEMA) to deal with the problem. They finally implemented their proposed model and algorithm on several test problems for validation. Amiri et al. [54] addressed GVRP and presented a bi-objective model to minimize transportation costs and greenhouse gas emissions and exploited the EC method and ALNS meta-heuristic algorithm to solve this problem. Asghari et al. [55] studied EVRP, considering recovering a pre-determined schedule of an EV in case of an unpredicted disruption to determine the optimal vehicle velocity and the policy for battery recharging. They solved the model by using a PSO-based meta-heuristic algorithm. The findings indicate that the application of the recovery actions can greatly decrease disruption costs. Ma et al. [56] incorporated congestion and energy consumption into EVRP with the objective of minimizing cost and solved this problem by applying the ALNS meta-heuristic algorithm. The performance of the algorithm was also examined using test problems. Zhang et al. [57] studied the long-term bus electrification that simultaneously optimizes the charging infrastructure deployment and bus fleet transition. Han et al. [58] proposed a MIP model for the routing problem of electric trucks, considering multiple charging alternatives with the objective of minimizing total transportation costs. They applied the ALNS algorithm to solve the model on test problems, and the findings demonstrated the efficiency of the algorithm. Cai et al. [59] investigated some of the main issues of EVRP with backup batteries, battery swapping stations, and time windows to simultaneously optimize different tasks by exploiting a novel meta-heuristic algorithm. They also validated their model and algorithm using 30 numerical examples as well as four real-world problems. Abid et al. [60] attempted to iterate real-world parameters such as energy consumption, heterogenous fleets, and infrastructure data in order to minimize the total number of vehicles used, distance traveled, travel time, and energy consumption. In addition, several studies have been conducted on EVs and energy consumption [61,62,63,64].

A comprehensive study of the literature demonstrates that despite several research studies conducted on EVRP, some gaps still exist in this field. As a result, this paper seeks to contribute to the existing literature by filling these gaps. The primary contributions of the present study can be mentioned as follows:

- Presenting a multi-objective mathematical programming model for the Capacitated Electric Vehicle Routing Problem (CEVRP), taking both soft and hard time windows as well as the possibility of EV charging into consideration,
- Proposing a hybrid of Non-dominated Sorting Genetic Algorithm (NSGA-II) and Teaching–Learning-Based Optimization (TLBO) meta-heuristic algorithms to solve this problem,
- Comparing the performance of the proposed hybrid NSGA-II-TLBO meta-heuristic algorithm with other well-known meta-heuristic algorithms by solving different test problems based on six criteria,
- Performing statistical analysis for determining the meaningful difference between the performance of the algorithms according to six criteria.

## 3. Problem Definition

**Figure 1:**
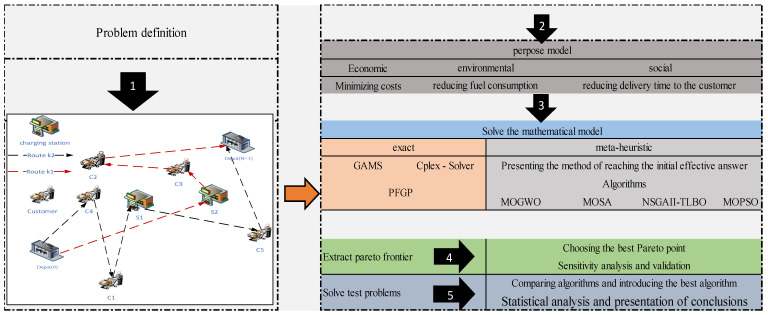
The conceptual model of the research.

Recently, sustainability has gained special importance in all logistics and transportation operations, so if a logistics company does not consider the sustainability dimensions, it will be removed from the global competition. On the other hand, maximizing customer satisfaction with on-time service delivery has recently attracted much more attention. Sustainable logistics networks seek to reduce costs (economic dimension), maximize customer satisfaction by providing timely services (social dimension), and reduce pollution in transportation (environmental dimension). Hence, the Vehicle Routing Problems (VRPs) are extremely important in sustainable logistics networks since VRPs deal with finding the shortest routes, which include all aspects of sustainability. Therefore, this study investigates an EVRP in which EVs render services to customers to decrease pollution. EVs are employed for the delivery of commodities with diverse capacities according to the number of goods to be shipped to customers. The usage of similar EVs with the same loading capacities for different volumes of commodities leads to an increase in logistics and operational costs as well as environmental risks. This study deals with the design of a network that delivers customers’ demands using electric cars that need to visit multiple charging stations for recharging. Traditional logistics networks seek to reduce costs, do not pay attention to environmental and social aspects, and are no longer effective [12,21]. For this purpose, in this research, maximizing customer satisfaction by providing timely services (the social aspect) and reducing pollution in transportation (the environmental aspect) are addressed. The distribution network is depicted in Figure 1.

Figure 1 shows the schematic of the problem in the first stage. In the second stage, the objectives of the research, the third stage shows the solution methods, and the 4th and 5th stages show how to solve it.

## 4. Multi-Objective Mathematical Model

The proposed model includes the following assumptions: The set *C* represents the service (customer) points, and the set *R* denotes the charging stations. Also, there is only one depot. In addition, a graph of *A* = (*V*, *G*) is assumed in which $V=\left\{0\right\}\cup C\cup R$ denotes the present nodes containing the depot, charging stations, and customers. Moreover, *A* shows the set of arcs. EVs with various capacities begin their tours at the depot. Each vehicle consumes energy, whose amount is computed based on the distance traveled.

1. A group of customers that need to receive goods are included in a distribution network.
2. The EV fleet is heterogeneous.
3. The tour of each EV starts at the depot with the total amount of goods that must be delivered to the customers, and each EV has a full charge while departing the depot.
4. Partial delivery is not allowed.
5. Each customer must be serviced (receive goods) once with a certain fleet of identical EVs.
6. Each tour terminates in the depot, and a time window is assumed for the time to reach the depot and can go beyond the allowable time; however, this amount is taken into consideration in the environmental (second) objective function.
7. Every EV is chosen for one route.
8. A hard time window is assumed for the customer’s visiting time for rendering service, which must not go beyond the time interval.
9. A certain time interval is assumed for the vehicles’ accessibility.
10. There are various types of EVs with a defined capacity.
11. The vehicle load cannot exceed the vehicle capacity at any time during the tour.
12. The capacity of the depot is large enough.
13. Charging stations may be visited more than once by an EV.
14. Partial charging is also possible.
15. EVs are assumed to be charged at a fixed rate at charging stations.
16. The battery charge of the EV and its load must be zero when it returns to the depot.
17. The load flow is calculated for each arc.

Sets

i and j: Node

ne: number of EVs e 

${nF}^{\prime }$: number of charging stations

$F_{0}^{\prime }$: The set of initial depots and charging stations 

nc: number of customers V={1,2,…,N}

${nV}_{0}$: The set of initial depots and customers 

${nV}^{\prime }$: The set of charging stations and customers $V^{\prime }=V\cup F^{\prime }$

${nV}_{0}^{\prime }$: The set of initial depots, charging stations, and customers $V_{0}^{\prime }=V^{\prime }\cup \{0\}$

Parameters

${\omega c}_{i}$: The weight of each customer’s order

${CRV}_{e}$: The consumption rate of vehicle e

${DBN}_{ij}$: Distance between nodes *I* and *j*

${tnp}_{ije}$: Time needed for passing from node *i* to node j with vehicle *e*

${st}_{i}$: Service time for customer *i*

${es}_{in}$: Earliest start time *i* for customer service

${lt}_{in}$: Latest start time *i* for customer service

${CD}_{i}$: Customer demand *i*

g: Charging time rate

${cap}_{e}$: The energy capacity of EV *e*

${LCEV}_{e}$: The loading capacity of EV *e*

${RCV}_{e}$: Remained charge for each eVper node

${ELF}_{e}$: Penalty cost for the remaining charge for each EV 

${CTEV}_{ije}$: The cost of travel from node *i* to node *j* with EV *e*

${PEC}_{e}$: Penalty for early arrival to the customer for each EV

${PLC}_{e}$: Penalty for late arrival to the customer for each EV

LEN: A large enough number

Decision variables

${TAEV}_{ie}$: Time to arrive at the nod i with EV e

${RV}_{ie}$: The remaining load of EV n when reaching node i

$\beta_{ie}$: The remained charge of EV n when reaching node i

${EVX}_{ije}$: 1; if the EV n travels between nodes *i* and *j*, otherwise, 0.

${ALV}_{ij}$: The amount of load in each arc that the EV carries

${ET}_{ei}$: The earliest time that the EV reaches the customer

${LT}_{ei}$: The latest time that the EV reaches the customer

(1) $$Min\;O_{1}=\sum_{i}\sum_{j}\sum_{e}{CTEV}_{ijn}\times {EVX}_{ije}+\sum_{i}\sum_{e}{ELF}_{e}\times {RV}_{ie}+\sum_{i}\sum_{e}{PEC}_{e}\times {ET}_{ie}+\sum_{i}\sum_{e}{PLC}_{e}\times {LT}_{ei}\;$$

(2) $$Min\;O_{2}=\sum_{i}\sum_{j}\sum_{e}{CRV}_{e}\times {DBN}_{ij}\times {EVX}_{ije}+\sum_{i}\sum_{e}{RCV}_{e}\times \beta_{ie}$$

(3) $$Min\;O_{3}=\sum_{e}\left({TAEV}_{\left(”0”\right)e}\right)$$

Equation (1) shows the economic objective function, which consists of four parts. The first part calculates the cost of the route traveled by each vehicle *e*; the second part calculates the penalty associated with the remaining charge of each vehicle; and the third and fourth parts calculate the cost of arriving late or early to the customer. Equation (2) expresses the environmental objective function that seeks to minimize electricity consumption by reducing the distance traveled by EVs. Equation (3) shows the social objective function that minimizes the vehicle’s arrival time to the depot.

(4) $$\sum_{i\in {nV}_{0}^{\prime }}\sum_{e\in ne}{EVX}_{ije}=1\;\;\;\;\;\;\;\;\;\;\;\;\;\;\;\;\;\;\;\;\;\;\forall j\in nc$$

(5) $$\sum_{i\in {nV}_{0}^{\prime }}{EVX}_{jie}-\sum_{i\in {nV}_{0}^{\prime }}{EVX}_{ije}=0\;\;\;\;\;\;\;\;\;\;\;\;\;\;\;\;\;\;\;\;\;\;\forall j\in V^{\prime },e\in ne$$

(6) $$\sum_{j\in {nV}^{\prime }}{EVX}_{ije}\;\;\;\leq 1\;\;\;\;\;\;\;\;\;\;\;\;\;\;\;\;\;\;\;\;\;\;\;\;\;\;\;\;\;\forall i\in \{0\}$$

(7) $$\sum_{j\in {nV}^{\prime }}{EVX}_{jie}\;\;\;\leq 1\;\;\;\;\;\;\;\;\;\;\;\;\;\;\;\;\;\;\;\;\;\;\;\;\;\;\;\;\;\forall i\in \{0\}$$

(8) $${TAEV}_{in}+\left({tnp}_{ijn}+{st}_{i}\right){EVX}_{ije}-LEN\left(1-{EVX}_{ije}\right)\leq {TAEV}_{je}\;\;\;\;\;\;\;\;\;\;\;\;\;\;\;\;\;\;\;\forall i\in {nV}^{\prime },\;j\in {nV}_{0}^{\prime },\;i\neq j,e\in ne$$

(9) $${TAEV}_{ie}+{tnp}_{ije}{EVX}_{ije}+g\left({cap}_{e}-\beta_{ie}\right)-\left(LEN+g{cap}_{e}\right)\left(1-{EVX}_{ije}\right)\leq {TAEV}_{je}\;\;\;\;\;\;\;\;\forall i\in nF^{\prime },\;j\in V_{N}^{’},\;i\neq j,e\in ne$$

(10) $${et}_{je}\leq {TAEV}_{je}\leq {lt}_{je}\;\;\;\;\;\;\;\;\;\;\;\;\;\;\;\;\;\;\;\;\;\;\;\;\;\;\;\;\;\;\;\;\forall \;j\in V,e\in ne$$

(11) $$0\leq {RV}_{je}\leq {RV}_{ie}-{CD}_{i}{EVX}_{ije}+{LCEV}_{n}\left(1-{EVX}_{ije}\right)\;\;\;\;\;\;\forall i\in {nV}_{0}^{\prime },j\in {nV}_{N}^{\prime },e\in ne$$

(12) $$0\leq {RV}_{0e}\leq {LCEV}_{e}\;\;\;\;\;\;\;\;\;\;\;\;\;\;\;\;\;\;\;\;\;\;\;\;\;\;\;\;\;\;\;\;\;\;\;\;\;\;\forall e$$

(13) $$0\leq \beta_{je}\leq \beta_{ie}-{CR}_{e}{DB}_{ij}{EVX}_{ije}+{cap}_{n}\left(1-{EVX}_{ije}\right)\;\;\;\;\;\;\;\;\;\;\;\;\;\;\;\;\;\;\;\;\;\;\;\;\;\;\forall \;i\in V,j\in V_{N}^{\prime },i\neq j,e\in ne$$

(14) $$0\leq \beta_{je}\leq {cap}_{e}-{CR}_{e}{DB}_{ij}{EVX}_{ije}\;\;\;\;\;\;\;\;\;\;\forall \;i\in nF_{0}^{\prime },j\in {nV}_{N}^{\prime },e$$

(15) $$\;\sum_{i\in V}{ALV}_{ih}-\sum_{j\in V}{ALV}_{hj}={\omega c}_{h}\;\;\;\;\;\;\;\;\;\;\;\;\;\;\;\;\forall \;h\in V$$

(16) $$\sum_{n}{\omega c}_{h}\times {EVX}_{ijn}\leq {ALV}_{ij}\;\;\;\;\;\;\;\;\;\;\;\;\;\;\;\;\;\;\;\;\;\;\;\;\;\;\forall \;\;i,j\in V$$

(17) $${ALV}_{ij}\leq \sum_{e}{{[cap}_{e}-\omega c}_{i}]\times {EVX}_{ije}\;\;\;\;\;\;\;\;\;\;\forall \;\;i,j\in V$$

(18) $$\sum_{i,j\in V}{[\omega c}_{i}]\times {EVX}_{ije}\;\;\leq {cap}_{e}\;\;\;\;\;\;\;\;\;\;\;\;\;\;\;\;\;\;\;\;\;\forall \;\;e\in ne$$

(19) $${ET}_{ni}=max\left\{{es}_{in}-{TAEV}_{in},0\right\}\;\;\;\;\;\;\;\;\;\;\;\;\;\;\;\;\forall \;i\in V,e\;\in ne$$

(20) $${LT}_{ei}=max\left\{{TAEV}_{ie}-l_{je},0\right\}\;\;\;\;\;\;\;\;\;\;\;\;\;\;\;\;\;\;\;\;\forall \;\;i\in V,e\;\in ne$$

Constraints (4)–(7) represent the visited nodes and ensure that each customer is served by only one EV at a time so that customer demand is met. In fact, these restrictions determine the EV’s route. Constraint (8) represents the time to reach the nodes. Constraint (9) indicates the arrival time to the following nodes when EV is in the node of the charging station. Constraint (10) deals with the assumed hard time window associated with the arrival time to the customer node since each customer must be served at a determined time interval. Constraint (11) indicates the amount of the EV load in the following nodes in such a way that when the necessary load (that is less than or equal to the loading capacity of EV) is loaded at the pickup depot, it is decreased when visiting each customer. Constraint (12) dictates that the vehicle load in the pickup depot must be equal to the maximum loading capacity of an EV. Constraint (13) displays the amount of EV charge in the successive nodes. Constraint (14) represents the amount of vehicle charge in the consecutive nodes. In other words, this constraint states that the energy level of an EV is at its maximum when it leaves the initial depot or charging station. Constraints (15) to (18) control the load flow of the vehicle. Constraints (19) and (20) are related to the hard time window for satisfying customer demand.

## 5. Solution Approaches

Goal programming (GP) is one of the most practical multi-objective approaches. Decision makers assign specific values to objective functions as their expectation levels or goals in the GP approach and then try to sum up the deviations from the expectation levels to minimize. In this method, the ideal of the objective functions must be expressed with a specific value, and since there is uncertainty in real problems, it is almost impossible to determine a certain value for the desired goal. One of the most effective approaches to dealing with uncertainty in the decision-making process is the fuzzy sets theory. Therefore, the fuzzy goal programming (FGP) approach, as a combination of fuzzy sets theory and GP, has been introduced as a practical approach [65].

PFGP is a new development of FGP that was first introduced by Mirzaei et al. in 2018. In this method, goals are prioritized, and the degree of achievement of the most important goal should be higher than the degree of achievement of other goals. These priorities are added to the model through some equations as priority constraints. PFGP can be implemented on multi-objective problems with uncertainty in objectives and objectives with varying importance. The variables and parameters of the PFGP method are described as follows [66]:

$\mu_{o}$: The degree of achievement of the goal
$U_{o}$: The upper limit for the goal
$L_{o}$: The lower limit for the goal
$d_{o}^{+}$: Positive changes from the expected level
$d_{o}^{-}$: Negative changes from the expected level
${go}_{o}$: The expected goal of the objective function

The general formulation of PFGP is described below:

(21) $$MaxZ=\sum_{o}\mu_{o}$$

(22) $$\mu_{o}+\frac{1}{U_{o}-{go}_{o}}\times d_{o}^{+}\leq 1\;\;\;\;\;\;\;\;\;\;\;\;\;\;\;\;\;\;\;\;\;\;\forall o$$

(23) $$g_{c}\left(x\right)=\left(\leq \geq \right)0\;\;\;\;\;\;\;\;\;\;\;\;\;\;\;\;\;\;\;\;\;\;\forall c$$

(24) $$f_{o}-d_{o}^{+}\leq {go}_{o}\;\;\;\;\;\;\;\;\;\;\;\;\;\;\;\;\;\;\;\;\;\;\forall o$$

(25) $$\mu_{o}+\frac{1}{{go}_{o}-L_{o}}\times d_{o}^{-}\leq 1\;\;\;\;\;\;\;\;\;\;\;\;\;\;\;\;\;\;\;\;\;\;\forall o$$

(26) $$\mu_{o}\geq \mu_{o^{\prime }}\;\;\;\;\;\;\;\;\;\;\;\;\;\;\;\;\;\;\;\;\;\;\forall o\neq o\prime$$

(27) $$f_{o}+d_{o}^{-}\geq {go}_{o}\;\;\;\;\;\;\;\;\;\;\;\;\;\;\;\;\;\;\;\;\;\;\forall o$$

(28) $$\mu_{o},d_{o}^{-},d_{o}^{+}\geq 0$$

Equation (21) represents the goal of the PFGP method for maximizing the degree of achievement of all goals. Constraints (21)–(28) represent the system constraints and $f_{o}$ represents the objective function *o*.

### 5.1. Solution Representation Scheme

In this research, meta-heuristic algorithms and MATLAB software (2020b) were used, but first, the way to reach a feasible solution in MATLAB software should be presented. In this research, in order to obtain an initial feasible solution, a solution representation scheme is used to determine the sequence of visits and assign customers to vehicles.

**Figure 2:**
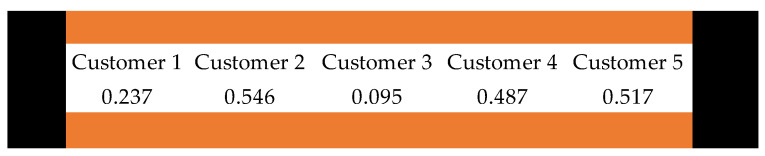
An example of the solution chromosome.

**Figure 3:**
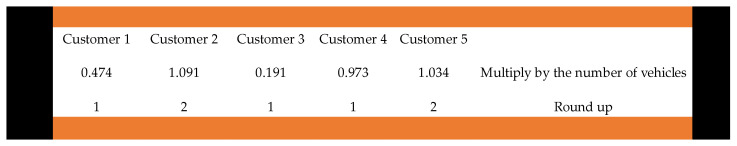
An example of the solution chromosome.

**Figure 4:**
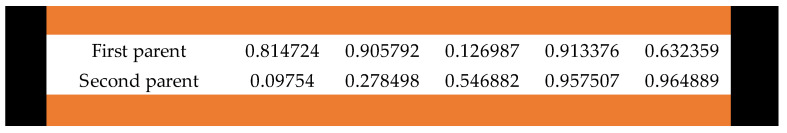
An example of a crossover operator.

**Figure 5:**
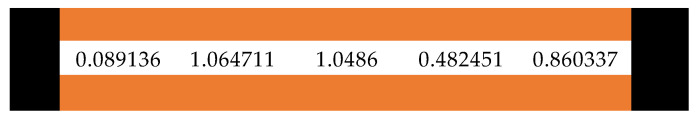
An example of a crossover operator.

**Figure 6:**
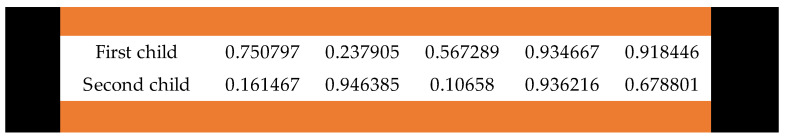
An example of crossover operator.

For this purpose, a random vector of numbers between zero and one with the dimension of *l* is formed, where *l* is the number of customers. Then, we multiply the resulting vector by *K* (the number of EVs) and round the result upwards. In this way, customers are allocated to EVs. In order to determine the sequence of customers for each EV, random numbers between zero and one are sorted in ascending order in the initial vector, and customers are visited based on the sorting (Figure 2, Figure 3, Figure 4, Figure 5 and Figure 6). For example, if we have 5 customers and 2 EVs, a random vector is formed as follows:

By multiplying the above vector by the number of EVs and rounding up, we will have:

Now, based on the sorting of the primary vector in ascending order, the order of their visits will be as follows:

The first vehicle: Customer 3, Customer 1, and Customer 4
The second vehicle: Customer 5 and Customer 2.

Considering that all the variables are related to the departure of EVs to the customers, if an EV has visited the customer, its needed goods are delivered and its demand is met.

Guaranteeing the solution feasibility

While updating chromosomes based on mutation and crossover operators, chromosomes may be generated whose values are infeasible and do not satisfy the model constraints. In order to resolve this issue, we use a penalty function in which the violation value is multiplied by a large number and added to the objective function values. This problem has one penalty function. The penalty function of this problem is the amount of violation of the maximum available time of EV (denoted by *r*). According to this penalty function, we obtain the violation amount of the maximum available time of EV, multiply this amount by a large number, and add it to the objective function values. If the violation value is equal to zero, no penalty is considered, but if there is a violation, the value of a large number is added to the objective function values and causes their values to deteriorate.

(29) $${CV}_{1}=\max\left(0\;.\;\frac{T_{k}}{r}-1\right)$$

In the above equation, ${CV}_{1}$ denotes the violation amount, $T_{k}$ represents the time to return to the depot, and *r* denotes the maximum available time for the EV *k*. Based on the violation functions, the values of the objective functions are obtained by Equations (30)–(32):

(30) $${Obj}_{1}={OFV}_{1}+M\left({CV}_{1}\right)\;$$

(31) $${Obj}_{2}={OFV}_{2}+M\left({CV}_{1}\right)\;$$

(32) $${Obj}_{3}={OFV}_{3}+M\left({CV}_{1}\right)$$

Crossover operator

The arithmetic crossover is used to create new generations. In this type of crossover operator, if $x_{1}$ is the first parent and $x_{2}$ is the second parent, and each has *n* members, a random vector (α) between $\left[-\delta ,1+\delta \right]$ with the length of *n* is formed, where δ is the parameter. The first child $\left(y_{1}\right)$ and the second child $\left(y_{2}\right)$ are obtained by using Equations (33) and (34):

(33) $$y_{1}=\alpha .x_{1}+\left(1-\alpha \right).x_{2}\;$$

(34) $$y_{2}=\left(1-\alpha \right).x_{1}+\alpha .x_{2}$$

Suppose that the first and second parents are displayed as the following vectors (shown in Figure 4):

Considering the value of 0.1 for parameter *δ*, a random solution for the vector α is obtained as follows (depicted in Figure 5):

Using the aforementioned equations, the first and second children are obtained as follows (displayed in Figure 6):

Mutation operator

The mutation operators are used in the optimization algorithms for diversifying the solutions and preventing convergence to the local optimum. The mutation operators are applied to the solutions as well as to the best solution that has the highest fitness value. In each step, the best population or solution is saved as the solution. The mutation operator used in this research is that μ members are selected from each chromosome, and those members are added with a continuous random number between [−1, +1].

### 5.2. Multi-Objective Simulated Annealing (MOSA) Algorithm

The Simulated Annealing (SA) algorithm was introduced by Kirkpatrick et al. in 1983 [67]. This probabilistic discrete meta-heuristic algorithm has been widely employed to solve numerous combinatorial optimization problems due to its simplicity and efficiency. The primary notion of SA originates from the metal annealing processes. The gradual and step-by-step annealing technique is employed by metallurgists to attain a state where solids are well-adjusted and energy is downgraded. The aim is to achieve the crystal size of the solid material at the highest level in the annealing process. In the SA algorithm, the temperature of the material is raised to the highest level, and then the temperature is gently lowered. The annealing process is simulated to obtain a globally minimum response. The SA algorithm begins with a random initial solution, and the temperature of the system is equal to the beginning temperature (T = T_0_). At each iteration, a neighborhood solution is found. The value of the objective function of the consequent solution is compared to the objective function value of the present solution. If the new solution is better than the previous solution, it will be swapped with the previous solution; otherwise, it will be swapped with the previous solution with a probability computed by the Baltsman’s function, which avoids being trapped in a locally optimal solution. A sufficient number of iterations may be considered the stopping criterion for the SA algorithm. When the algorithm stopping criterion is satisfied, the system temperature diminishes. This procedure carries on until the SA algorithm gets to the endpoint [68,69,70]. The extended version of this algorithm, known as Multi-Objective Simulated Annealing (MOSA), is exploited to solve optimization problems with conflicting objectives.

### 5.3. Multi-Objective Grey Wolf Optimization (MOGWO) Algorithm

The Grey Wolf Optimization (GWO) algorithm is a meta-heuristic algorithm inspired by the hierarchical structure and social behavior of hunting gray wolves. This population-based algorithm has a simple process and can easily be generalized to large-scale problems. The extension of this algorithm, known as Multi-Objective Grey Wolf Optimization (MOGWO), is used for solving optimization problems with conflicting objectives. MOGWO considers an external archive with a fixed size to store and retrieve Pareto optimal solutions. This archive is utilized to define social hierarchy and simulate the hunting behavior of gray wolves in multi-objective search spaces. For more details about this algorithm, we refer to Mirjalili et al. [71].

### 5.4. Non-Dominated Sorting Genetic Algorithm II (NSGA-II)

The Genetic Algorithm (GA) as a population-based method was proposed by Deb et al. [72]. The Non-dominated Sorting Genetic algorithm (NSGA-II) has been extensively applied to solve multi-objective optimization problems. For more details about NSGA-II, we refer to Goldberg and Holland [73]. In this algorithm, chromosomes are used to present solutions. At each iteration, the next offspring is generated using the crossover and mutation operators, and then the obtained solutions are sorted regarding the non-domination theory. In order to maintain diversity in the population, a crowding distance measure is allocated to each member of the population. If the number of non-dominated solutions goes beyond the population size, the NSGA-II algorithm chooses the least crowded solutions according to the crowding distance measure and ignores the remaining non-dominated solutions. Therefore, both diversity and convergence of the Pareto solutions are assured [74].

The fundamental steps of the NSGA-II algorithm can be described as follows:

1. Generating the initial population,
2. Calculating the fitness values (according to the objective functions),
3. Sorting non-dominated population and computing the swarm distance,
4. Implementing the crossover and mutation operators for generating the next population (offspring),
5. Combining the initial population with the next population generated by the crossover and mutation operators,
6. Swapping the initial population with the fittest (best) population members generated in the previous steps,
7. All the aforementioned steps are iterated to achieve the optimal conditions.

### 5.5. Teaching–Learning-Based Optimization (TLBO) Algorithm

The TLBO algorithm was inspired by the teacher’s educational influence on students’ output (learning) and is appropriate for engineering applications. A teacher is generally defined as someone with a higher level than the students and who is capable of sharing knowledge with them. Hence, at each iteration, the teacher is a solution with the best objective function value. However, the teacher may be different at any iteration. The teacher literally lectures the lessons and assesses the students based on their scores. For more information about the TLBO algorithm, we refer to Rao et al. [75] and Rao et al. [76].

### 5.6. Hybrid of Non-Dominated Sorting Genetic Algorithm (NSGA-II) and Teaching–Learning-Based Optimization (TLBO) Algorithms

**Figure 7:**
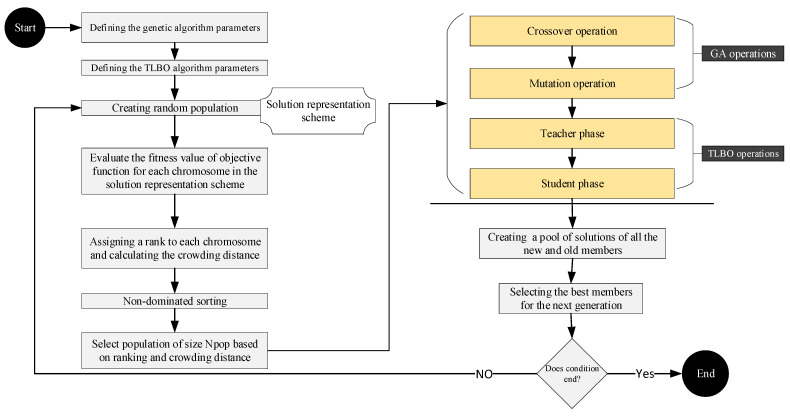
Flowchart of the NSGA-II-TLBO algorithm.

The Teaching–Learning-Based Optimization (TLBO) algorithm includes two phases for solution search, leading to a more profound exploration of finding a globally optimal solution. The combination of TLBO and NSGA-II increases the diversification of solutions and eludes trapping in a local optimum. This hybrid algorithm begins with a random initial population. At each iteration, the NSGA-II crossover and mutation operators are first applied to the initial population to generate the next population; subsequently, the TLBO operators improve the obtained solutions. As a result, both intensification and diversification of the meta-heuristic algorithm enhance. The process of the proposed algorithm is shown in Figure 7.

As depicted in Figure 7, first the parameters of the NSGA-II and TLBO algorithms are defined and set, then the initial solutions for the given problem are generated by using the method described in the “Solution representation scheme” section. Subsequently, the operators of the two algorithms are implemented on the chromosomes, and the solutions are ranked. Finally, the set of Pareto solutions is obtained by using the non-dominated sorting function [45,46,48,50,54,77,78].

### 5.7. Multi-Objective Particle Swarm Optimization (MOPSO) Algorithm

The particle swarm optimization (PSO) algorithm is one of the most important intelligent optimization algorithms in the field of swarm intelligence. This algorithm was inspired by the social behavior of animals such as fish and birds that live together in different groups [43,44,46,48,52,70,71].

## 6. A Numerical Example for Validating the Proposed Model and Methodology

In order to validate the proposed mathematical programming model and compare the solutions of the proposed meta-heuristic algorithms, a numerical example was considered and solved by the FPGP exact method along with the MOSA, MOGWO, NSGA-II-TLBO, and MOPSO meta-heuristic algorithms. The data for the numerical example are given in Table 1. According to Table 1, this problem comprises 7 customers, 2 charging stations, and 2 heterogeneous vehicles.

The results of solving the mathematical model using the FPGP method are presented in Table 2. According to Table 2, improving the value of the economic (first) objective function leads to the deterioration of the value of the environmental (third) objective function, as well as the deterioration of the value of the social responsibility (third) objective function dealing with customer service time, which demonstrates the conflict between the objective functions. Table 2 shows the range of epsilon that was achieved from the upper limit and lower limit of each objective. For example, the minimum value of the first objective function is 7125.3, and the maximum value of this objective function is 31,247.1. Also, the minimum value of the second objective function is 22.054, and the maximum value of this objective function is 41.08. In addition, the minimum value of the third objective function is 23, and the maximum value of this objective function is 117.

**Figure 8:**
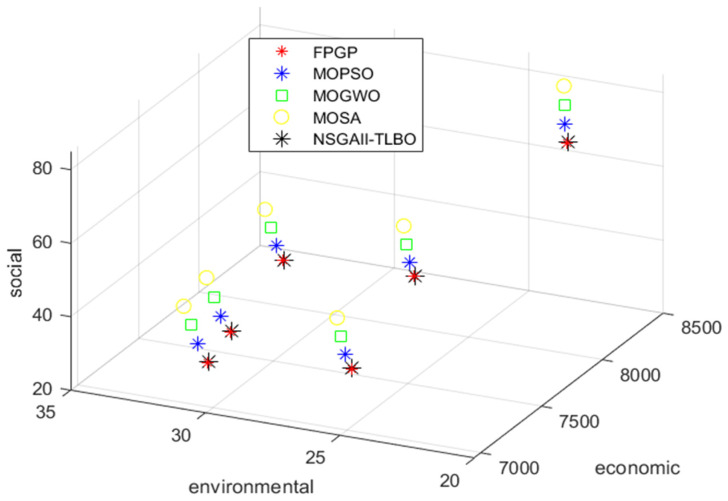
Three-dimensional Pareto front obtained by FPGP, MOSA, MOGW, MOPSO, and NSGA-II-TLBO.

In the following, by choosing six ideals in the acceptable ranges for economic, environmental, and social goals, a three-dimensional Pareto front was obtained from the FPGP method, which is presented in Table 3. To compare the Pareto front obtained from the FPGP method with the Pareto fronts obtained from the meta-heuristic algorithms, the aforementioned problem was implemented in MATLAB version 2021a software, and the results are presented in Figure 8. The results show that the Pareto fronts obtained from all meta-heuristic algorithms are close to the Pareto fronts obtained from the FPGP exact method.

**Figure 9:**
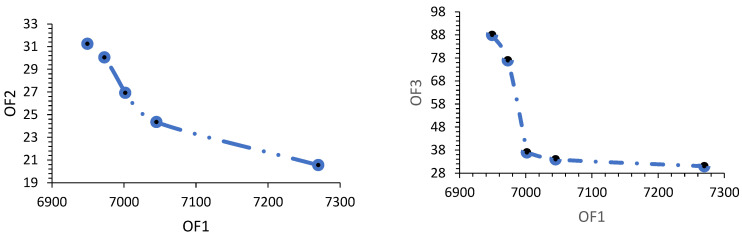
Relationship between economic, environmental, and social objectives.

The results of Pareto point 6 are displayed in Figure 8 and Figure 9.

According to Figure 9, to reduce the arrival time for customers and electricity consumption, higher costs are incurred. Similarly, if the focus of the decision-makers is on cost minimization, they should be indifferent to environmental and social objectives. This issue is always raised as one of the challenges in multi-criteria decision-making, and managers should make the best decision and select one of the Pareto solutions, considering the importance of the three sustainability goals to the company, which can affect the business future of the company.

**Figure 10:**
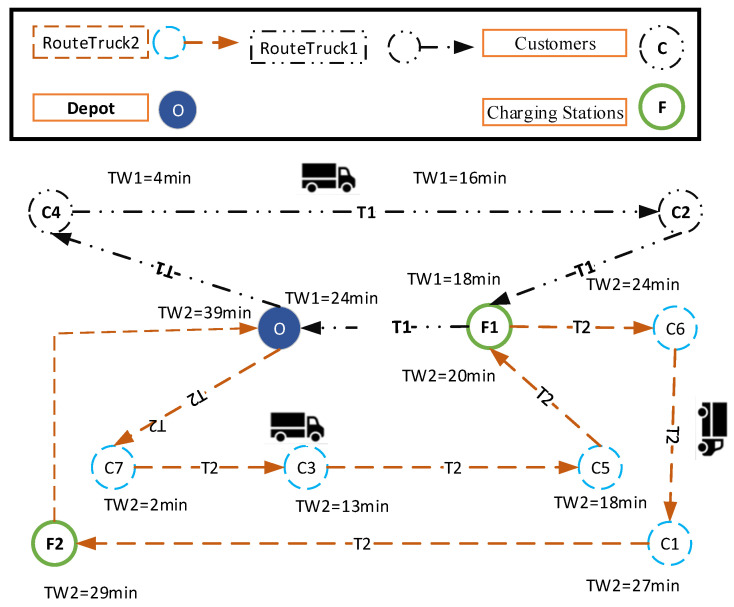
Route of EVs for the selected Pareto point 4.

The route of vehicles 1 and 2 and the arrival time for each customer at the selected Pareto point are depicted in Figure 10. As seen in this figure, the order of meeting customers is based on soft and hard time windows, and the vehicle continued to meet customers as much as possible (as long as it has charge), and if necessary, it was recharged. The route of the first vehicle is [O-C4-C2-F1-O] and the route of the second vehicle is [O-C7-C3-C5-F1-C6-C1-F2-O] so that all the customers’ demands were met. It should be noted that Figure 10 shows the moving path of the truck, the black path is for truck number 1 and the brown path is for truck number 2.

### 6.1. Comparison of the Proposed Methods for Solving the Mathematical Programming Model

In this section, 20 different problem examples (according to Table 4) have been created to compare the performance of the proposed algorithms in terms of three indexes and solve them using the provided solution methods. These examples have different parameters, including the number of vehicles (2 to 15), the number of customer nodes (5, 10, 15, and 100), charging stations (1 to 10), and coordinates of points. The values of the problem parameters are generated by the random distribution functions shown in Table 1 [13].

The first three test problems were solved using the MOSA, MOGWO, MOPSO, and NSGA-II-TLBO meta-heuristic algorithms, as well as the FPGP exact method (the CPLEX solver). It should be noted that each test problem was run 30 times by each algorithm, and the average for each objective function value is reported. The findings are provided in Table 5. As can be seen in Table 5, the values of each objective function are close to the values obtained from the FPGP method (the CPLEX solver) as the exact method.

It can be concluded from Table 5 that the values obtained by the meta-heuristic algorithms are close to the values obtained by the FPGP exact method (the CPLEX solver); hence, the proposed meta-heuristic algorithm is reliable for solving large-sized test problems.

### 6.2. The Criteria for the Performance Comparison of the Meta-Heuristic Algorithms

The performance of these three meta-heuristic algorithms is compared in this section. If a single-objective optimization problem (maximization problem) is in hand, it is clear that any feasible solution with a higher objective function value is better. However, in the case of MODM, the evaluation method is different. There are different ways to evaluate the performance of algorithms. One of these approaches is to fully investigate the solution space, obtain all the non-dominated points, and compare the solutions obtained by the algorithms with them. However, in practice, this is suitable only for small-sized problems, not for large-sized problems. To this end, the well-known indices for comparing the performance of multi-objective meta-heuristic algorithms are applied. Algorithm evaluation criteria are often divided into two categories. The first category deals with the convergence and quality of the solutions, and the second category focuses on the diversification of the solutions in the solution space. In this study, four indexes of CPU time, Mean Ideal Distance (MID), Multi Objective Coefficient of Variation (MOCV), and Hypervolume Indicator (HV) are employed to compare the performance of the MOGWO, MOSA, MOPSO, and NSGA-II-TLBO meta-heuristic algorithms [79,80].

Mean Ideal Distance (MID)

The MID index measures the distance between the Pareto solutions and the ideal point of those solutions using Equation (30). In this equation, $c_{i}$ denotes the distance of the objective functions of the *i*t Pareto point from the objective functions of the ideal point. The lower the value of the MID index, the higher the performance of the algorithm [81].

(35) $$MID=\frac{\sum_{i=1}^{NOS}c_{i}}{NOS}$$

Multi Objective Coefficient of Variation (MOCV)

This index is actually calculated by dividing the Mean Ideal Distance (MID) index by the diversity index. It is clear that the lower values of this criterion are desirable for comparing meta-heuristic algorithms [82].

Hypervolume Indicator (HV)

The HV indicator (or s-metric) is a performance metric for measuring the quality of a non-dominated approximation set, which was presented by Zitzler and Thiele in 1998 and described as the size of the space covered or the size of the dominated space [83].

## 7. Performance Evaluation of the Proposed Meta-Heuristic Algorithms

20 test problems, which are presented in Table 4, were randomly generated to compare the performance of the MOSA, MOGWO, MOPSO, and NSGA-II-TLBO meta-heuristic algorithms in terms of the aforementioned four indexes. For each meta-heuristic algorithm, each test problem was run 30 times, and the average value was reported as the ultimate solution. The values of the indices are provided in Table 6 and Table 7.

**Figure 11:**
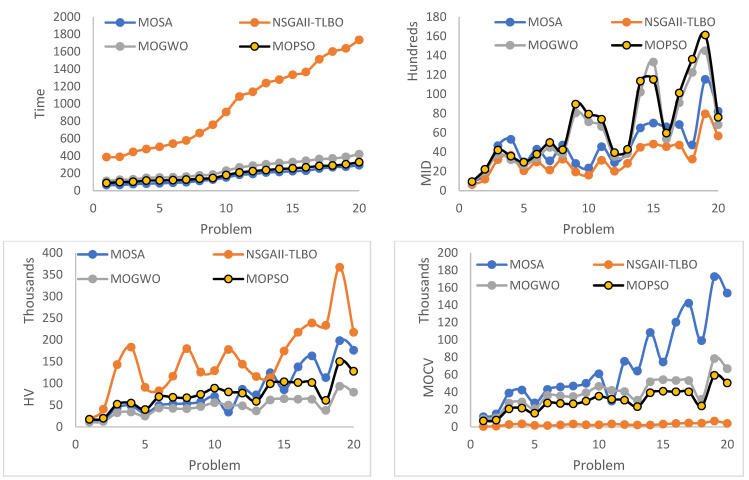
Comparison of algorithms in terms of the four indexes.

According to Table 6 and Table 7, the algorithms were compared in terms of the aforementioned indexes. As it can be seen in Figure 11, the NSGA-II-TLBO algorithm outperforms according to the MID, MOCV, and HV indexes. On the other hand, in terms of time index, the MOSA algorithm performs better than other algorithms. The performance of the NSGA-II-TLBO meta-heuristic algorithm is the worst in terms of time index, which was predictable as the steps of this algorithm increased because of the combination with the TLBO algorithm, and it requires more computational time than other algorithms.

## 8. Statistical Analysis

The ANOVA test is employed to analyze and compare the performances of the algorithms in terms of the four aforementioned indexes. For this purpose, the data must follow the normal distribution function. Therefore, the Minitab software was used, and the normality test was performed for the data (the outputs of the algorithms) of each index. 20 test problems (presented in Table 6 and Table 7) were used to evaluate the performance of the algorithms. The aim is to find a meaningful difference in the performance of the NSGA-II-TLBO, MOSA, MOPSO, and MOGWO algorithms. To this end, the one-way ANOVA test is performed to investigate the equality of the normal population means. If α≥P−value, the hypothesis $H_{0}$ (no meaningful difference between the means of the algorithms) will be rejected; otherwise, the hypothesis $H_{0}$ will not be rejected. For this purpose, α=0.05 [79,80,81].

**Figure 12:**
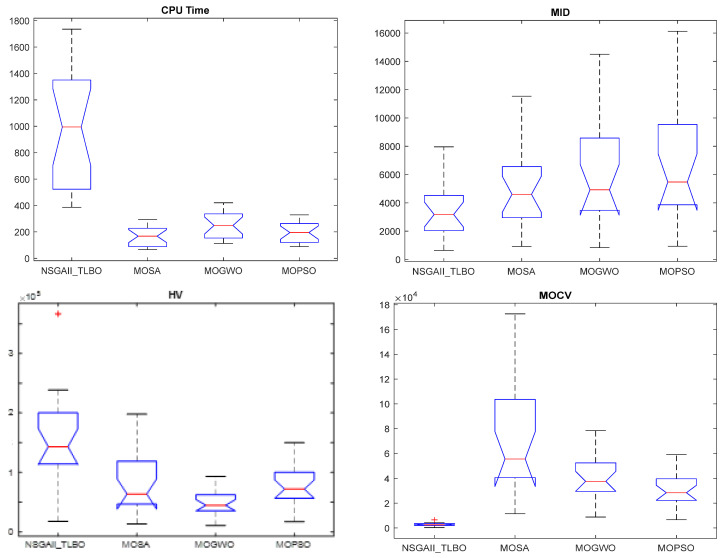
The results of the statistical analysis.

The MATLAB software was used to perform the statistical analysis for the algorithm outputs. The obtained *p*-values for the MOCV, MID, time, and HV indexes are equal to 0.004, 0.001, 0.002, and 0.034, respectively (displayed in Figure 12), which indicates that there are meaningful differences in terms of all indexes (time, MID, MOCV, and HV). NSGA-II-TLBO outperformed other algorithms according to the MID, MOCV, and HV indexes; however, the MOSA algorithm has the best performance in terms of the time index.

## 9. Discussion

The presence of vehicles at all customer points and the time to meet customer demand in the interval between the earliest and the latest allowed time indicate the validity of the proposed mathematical model.

The three objective functions of the model are related to each other. The economic objective function is in contradiction with the environmental and social objective functions, so increasing costs leads to better values for the environmental and social objective functions.

The sensitivity analysis performed on the two parameters of the vehicle charging time and the allowed time to return to the depot shows that the longer the vehicle charging time, the better solutions are obtained for the economic and social objective functions. Also, the longer the allowed time to return to the depot, the better the environmental objective function value because the vehicles have a better route and a better opportunity to charge and load.

In general, from a practical point of view, the proposed mathematical model can be applied to the current sustainable rechargeable EV routing problems in order to find out the optimal number of vehicles and the desirable routes.

## 10. Conclusions

In this study, a sustainable multi-objective mathematical programming model was presented for the electric vehicle routing problem (EVRP), considering the soft and hard time windows as well as the limitations related to load calculation. Minimization of system costs, minimization of vehicle electricity consumption, and minimization of the vehicle’s arrival time to customers were considered as the economic, environmental, and social objective functions, respectively. The main goal of the proposed optimization model was to reach a logical balance between the three sustainability aspects. The proposed model was validated using the FPGP exact method and the CPLEX solver. Since the problem is NP-Hard, the MOGWO, MOSA, MOPSO, and NSGA-II-TLBO meta-heuristic algorithms were employed to solve the large-sized problems. To this end, several test problems were generated to solve the proposed model using the meta-heuristic algorithms.

The results show that decreasing costs leads to increased service time and electricity consumption. In addition, improving the value of the environmental objective function leads to incurring more costs. Also, increasing customer satisfaction requires a significant increase in costs.

Moreover, the results demonstrate that it is very important to consider time windows to provide better services to customers and avoid high penalty costs.

Finally, the performance of the meta-heuristic algorithms was compared based on four indexes: time, MID, MOCV, and HV. The results indicate that the MOSA algorithm performs better in terms of the time index, and this difference is meaningful. On the other hand, the NSGA-II-TLBO algorithm outperforms in terms of the MID, MOCV, and HV indexes. The following suggestions are made for further research:

- Considering multiple depots in EVRP,
- Considering the conditions of uncertainty to achieve results similar to reality,
- Using artificial intelligence to predict system costs in the future,
- Solving the mathematical model with exact methods such as the Augmented Epsilon Constraint (AEC) method.

## Figures and Tables

**Table 1:** The problem data.

	${CD}_{i}$	${es}_{ie}$	${lt}_{ie}$		e1	e2
D0	0	0	0	${CRV}_{e}$	4	5
1	2	12	20	${cap}_{e}$	25	48
2	5	10	47	${CRV}_{e}$	55	65
3	7	13	21	${CTEV}_{ije}={DBN}_{ij}\times 2.5$		
4	9	18	29	g	0.001	
5	5	12	41			
6	3	15	49	${ELF}_{e}=U\sim [1-4]$		
7	8	12	29	${LCEV}_{e}=U\sim [2-6]$		
F1	0	0	0	${ECEV}_{e}=U\sim [2-5]$		
F2	0	0	0	${\omega c}_{i}=U\sim [2-8]$		
${tnp}_{ije}={DB}_{ij}\times 1.54$						

**Table 2:** Conflict between objectives and upper and lower limits for each objective.

Objective	PayoffMat			Min			Max		
	O1	O2	O3	O1	O2	O3	O1	O2	O3
O1	7125.3	11,256.9	31,247.1	7125.3	22.054	23	31,247.1	41.08	117
O2	25.02	22.054	41.08						
O3	108	117	23						

**Table 3:** The Pareto front achieved by the FPGP method.

	O1	O2	O3
1	7125.3	23.07	61
2	7051.07	30.18	32
3	7949.2	21.12	79
4	7091.7	25.24	34
5	7401.2	31.14	29
6	6941.7	27.09	65

**Table 4:** The VRP benchmark test problems (Schneider et al., 2014) [13].

Problem	Number of Customers	Number of Vehicles	Number of Charging Stations
1	5	2	1
2	10	2	2
3	15	2	3
4	20	2	4
5	25	2	5
6	30	3	6
7	35	3	6
8	40	3	6
9	45	3	6
10	50	3	6
11	55	4	7
12	60	4	7
13	65	4	7
14	70	4	7
15	75	4	7
16	80	6	8
17	85	6	8
18	90	8	8
19	95	8	8
20	100	10	10

**Table 5:** Comparison of algorithms in terms of upper and lower bounds for each objective function.

Problem	FPGP	MOPSO	MOGWO	MOSA	NSGAII-TLBO
	O1				
1	4617.53	4945.46	4675.20	4863.44	4817.52
2	5656.48	6055.20	5724.30	5905.74	5837.88
3	7272.62	7796.81	7370.72	8108.23	7440.87
	FPGP	MOPSO	MOGWO	MOSA	NSGAII-TLBO
	O2				
1	15.290	15.491	15.987	15.686	15.316
2	17.208	17.645	18.210	17.645	17.430
3	26.765	26.806	27.665	27.309	27.042
	FPGP	MOPSO	MOGWO	MOSA	NSGAII-TLBO
	O3				
1	46.78	47.36	46.85	47.96	46.84
2	52.63	53.94	53.36	53.95	53.28
3	81.86	81.97	81.08	83.52	82.69

**Table 6:** Results of solving problems with the MOSA and NSGA-II-TLBO meta-heuristic algorithms.

MOSA					NSGA-II-TLBO			
	Time	MID	HV	MOCV	Time	MID	HV	MOCV
1	65.09	919.02	13,231.02	11,549.16	386.27	633.68	17,791.52	319.97
2	65.7	1712.6	16,977.4	14,819.32	389.94	1180.86	40,493.75	728.26
3	75.1	4656.17	44,545	38,882.66	445.68	3210.51	142,673.26	2565.9
4	80.66	5311.5	48,515.08	42,348.07	478.73	3662.38	183,035.93	3291.8
5	85.07	2978.64	31,523.15	27,516.08	504.83	2053.83	90,933.26	1635.39
6	91.23	4288.6	49,247.5	42,987.39	541.38	2957.07	82,993.78	1492.6
7	97.32	3087.81	52,539.83	45,861.22	577.52	2129.11	116,355.12	2092.59
8	111.77	4714.77	53,777.57	46,941.63	663.32	3250.92	179,499.66	3228.21
9	127.86	2813.16	57,346.39	50,056.79	758.76	1939.73	125,680.74	2260.3
10	152.51	2337.07	69,921.12	61,033.09	905.11	1611.46	128,844.43	2317.2
11	182.54	4548.1	33,858.19	29,554.3	1083.32	3136	177,516.44	3192.54
12	191.64	2915.71	86,182.17	75,227.11	1137.31	2010.44	143,633.2	2583.17
13	208.55	4087.96	73,498.75	64,155.94	1237.68	2818.72	115,628.28	2079.51
14	215.32	6498.74	124,063.35	108,293.02	1277.86	4481.01	112,985.93	2031.99
15	224.73	6998.01	85,199.74	74,369.56	1333.72	4825.26	174,065.79	3130.48
16	230.16	6609.68	137,592.96	120,102.8	1365.96	4557.5	217,056.48	3903.65
17	254.68	6846.62	162,772.63	142,081.76	1511.43	4720.87	238,465.43	4288.67
18	269.82	4731.44	113,299.81	98,897.69	1601.3	3262.42	233,045.34	4191.2
19	276.01	11,529.54	197,777.95	172,637.37	1638.02	7949.83	366,747.57	6595.76
20	292.38	8221.25	175,970.2	153,601.72	1735.16	5668.71	216,963.08	3901.97
AV	164.9	4790.32	81,391.99	71,045.83	978.67	3303.01	155,220.44	2791.56

**Table 7:** Results of solving problems with the MOGWO and MOPSO meta-heuristic algorithm.

MOGWO					MOPSO			
	Time	MID	HV	MOCV	Time	MID	HV	MOCV
1	112.08	833.57	10,615.48	8916.37	87.64	926.83	17,041.83	6736.64
2	125.59	1988.58	12,214.20	10,259.20	98.20	2211.06	19,608.38	7751.20
3	130.49	3778.24	32,419.18	27,230.18	102.03	4200.94	52,044.95	20,573.38
4	147.60	3208.35	33,951.44	28,517.18	115.41	3567.30	54,504.80	21,545.75
5	151.44	2643.60	24,686.61	20,735.28	118.41	2939.37	39,631.29	15,666.25
6	154.69	3398.37	43,113.38	36,212.66	120.96	3778.58	69,213.16	27,359.97
7	162.27	4477.97	42,312.53	35,540.00	126.89	4978.96	67,927.49	26,851.75
8	176.17	3802.77	41,583.71	34,927.83	137.75	4228.22	66,757.46	26,389.23
9	186.16	8056.84	46,576.49	39,121.47	145.56	8958.23	74,772.76	29,557.68
10	229.68	7127.11	55,443.25	46,569.02	179.59	7924.48	89,007.22	35,184.57
11	267.72	6650.01	50,001.34	41,998.14	209.34	7394.00	80,270.92	31,731.10
12	287.23	3541.16	48,298.59	40,567.93	224.60	3937.34	77,537.37	30,650.53
13	303.83	3844.19	36,491.05	30,650.30	237.57	4274.27	58,581.82	23,157.40
14	319.05	10,205.72	61,709.78	51,832.52	249.47	11,347.52	99,067.36	39,161.33
15	329.02	13,316.20	64,525.86	54,197.87	257.27	11,518.73	103,588.23	40,948.44
16	343.25	5356.00	63,463.48	53,305.53	268.39	5955.22	101,882.70	40,274.24
17	363.45	9095.35	63,350.50	53,210.63	284.19	10,112.93	101,701.32	40,202.54
18	370.43	12,242.12	37,840.02	31,783.36	289.65	13,611.76	60,747.45	24,013.47
19	388.43	14,496.30	93,281.12	78,350.57	303.72	16,118.13	149,751.22	59,196.68
20	419.89	6828.57	79,495.31	66,771.32	328.32	7592.54	127,619.82	50,448.13
AV	248.43	9244.55	47,068.66	39,534.87	194.25	10,278.82	75,562.88	29,870.02

## Data Availability

Data are provided in the article.
